# Severe Drug-Induced Dermatosis in a Geriatric Patient: A Case Report

**DOI:** 10.7759/cureus.87781

**Published:** 2025-07-12

**Authors:** Stephanie Saldaña Guerrero, Sofia C Lanzarin Quezada, Quitzia L Torres Salazar

**Affiliations:** 1 Dermatology, Unidad Médica de Alta Especialidad (UMAE) No. 71, Mexican Social Security Institute (IMSS), Torreón, MEX; 2 Dermatology, General Hospital No. 16, Mexican Social Security Institute (IMSS), Torreón, MEX; 3 Biomedical Sciences, Universidad Juárez del Estado de Durango, Durango, MEX

**Keywords:** adverse drug reaction reporting systems, drug-induced, herbal medicine pharmacovigilance, pharmacovigilance, skin diseases

## Abstract

Older adults are particularly vulnerable to adverse drug reactions (ADRs) due to age-related physiological changes and polypharmacy. Among these, drug-induced dermatoses are common but often underdiagnosed, especially when presentation is atypical or when patients use unregulated treatments. We report the case of a 71-year-old woman who developed erythroderma, pruritus, and desquamation following the use of prescribed medications alongside topical and herbal self-medication. Hospitalization was required after outpatient treatment failed. Skin biopsy revealed an acute inflammatory pattern consistent with pharmacodermia. Conservative treatment with systemic corticosteroids and topical emollients achieved full remission without complications. This case highlights the diagnostic complexity of ADRs in older adults, particularly in the context of incomplete medication histories and the use of natural products. It emphasizes the need for heightened clinical suspicion, thorough medication review, and pharmacovigilance to prevent escalation of cutaneous toxicity in the elderly.

## Introduction

Adverse drug reactions (ADRs) are a major cause of morbidity and hospitalization in older adults, primarily due to age-related physiological changes, polypharmacy, and chronic multimorbidity. Among the broad spectrum of ADRs, cutaneous manifestations deserve particular attention because they are frequent and often represent the earliest visible signs of systemic toxicity. However, they may be overlooked or misattributed, especially in geriatric populations. Among these, cutaneous ADRs (often referred to as drug-induced dermatoses) are common but can be diagnostically challenging, as they mimic a wide range of dermatologic and systemic conditions [1].

The elderly are particularly vulnerable to ADRs because of altered pharmacokinetics and pharmacodynamics, including reduced renal clearance, diminished hepatic metabolism, and changes in body composition that enhance drug accumulation and toxicity [2].

Hospital-based studies report ADR prevalence rates of up to 16% among older adults, with over half of the cases linked to frequently prescribed drugs like diuretics, antibiotics, and analgesics. Importantly, many of these reactions are preventable, emphasizing the need for cautious prescribing, early recognition, and prompt withdrawal of offending agents [3]. Severe drug-induced dermatoses such as Stevens-Johnson syndrome (SJS), toxic epidermal necrolysis (TEN), and erythroderma pose a particular risk in geriatric patients, especially when clinical presentations are atypical.

Herein, we present the case of a severe drug-induced dermatosis in a geriatric patient, highlighting the diagnostic and therapeutic challenges encountered. This report follows the CARE (CAse REport) guidelines [4] and aims to contribute to the limited but crucial literature on pharmacodermias in older adults, advocating for heightened pharmacovigilance in aging populations.

## Case presentation

A 71-year-old woman with no relevant family history of dermatologic or autoimmune disorders presented to the emergency department with a three-day history of progressive pruritic skin lesions. The symptoms began on her hands and neck and extended to her face and upper limbs, accompanied by facial edema and desquamation. She reported a prior episode in June 2024, with erythema and scaling on the arms, neck, and back, previously treated with methotrexate, folinic acid, loratadine, and emollients. She also admitted to self-medicating with topical agents (Vitacilina®, Barmicil®) and consuming unspecified herbal remedies.

**Figure 1:**
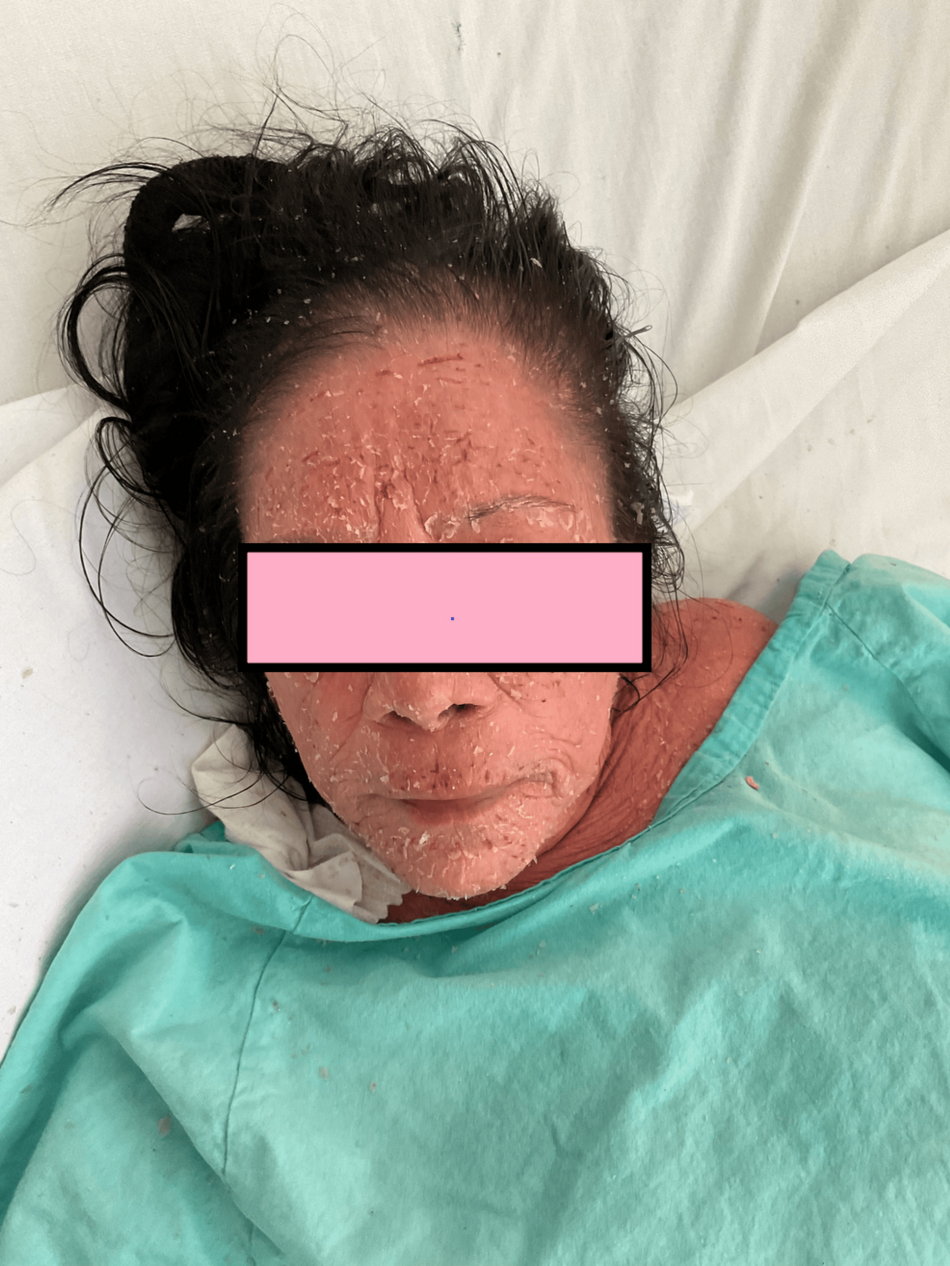
Facial view on admission showing diffuse erythema, edema, and marked desquamation, predominantly involving the periorbital and perioral regions, consistent with severe drug-induced dermatosis.

**Figure 2:**
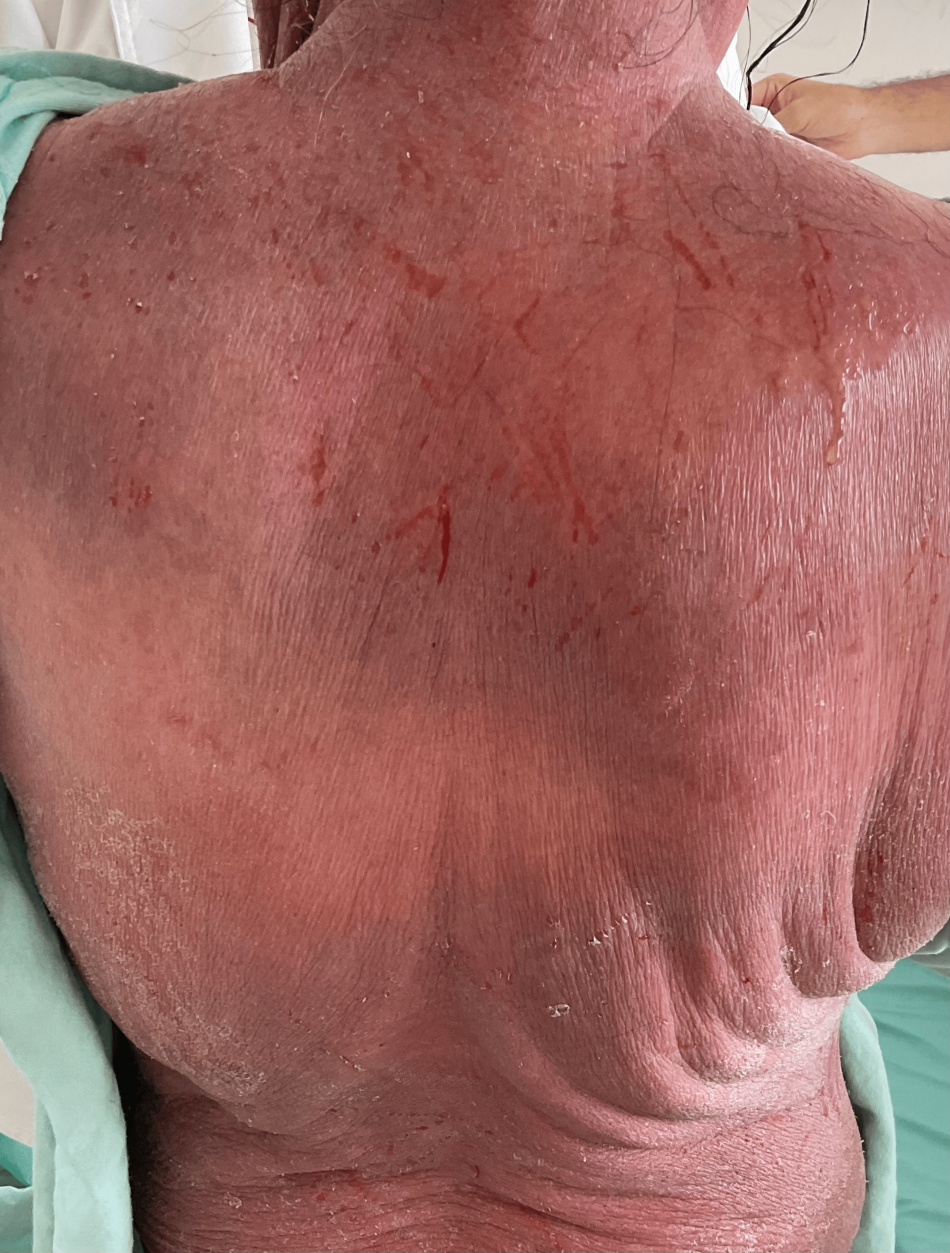
Extensive erythema and desquamation of the upper back with linear excoriations, indicating severe pruritus and active inflammation during the acute stage of drug-induced erythroderma.

Initial physical examination revealed diffuse erythema on the face, neck, and anterior arms, with mild edema and scaling. Mucous membranes, palms, and soles were spared (Figures 1, 2).

No vesicles or pustules were observed, and Nikolsky’s sign was negative. A presumptive diagnosis of contact dermatitis was made, and outpatient treatment was initiated with oral deflazacort (30 mg/day, tapered), loratadine (10 mg/day), and dicloxacillin (500 mg every six hours for seven days).

The patient returned days later with worsening symptoms: generalized erythema, pruritus, and desquamation extending to the trunk and upper limbs. The timeline is presented in Table 1.

**Table 1:** Clinical course and management timeline.

Day	Clinical events	Treatment/Findings
Before Day 0	Previous episode (June 2024) of erythema and scaling on arms, neck, and back	Treated with methotrexate, folinic acid, loratadine, and emollients
Day 0	Onset of pruritic lesions on hands and neck; spread to face and upper limbs	Self-medication with Vitacilina®, Barmicil®, and herbal remedies
Days 1-3	Facial edema and desquamation; outpatient treatment initiated	Oral corticosteroids and antibiotics prescribed (deflazacort, dicloxacillin)
Day 4	Worsening erythema and generalized desquamation; patient returned to the emergency department	Clinical deterioration despite outpatient therapy
Days 5-6	Hospitalization; lab tests revealed leukocytosis and hyperglycemia; skin biopsy performed	Biopsy: focal spongiosis and superficial perivascular infiltrate
Day 7	Conservative treatment maintained (systemic corticosteroids and emollients); marked clinical improvement	Lesions resolved completely without recurrence

**Figure 3:**
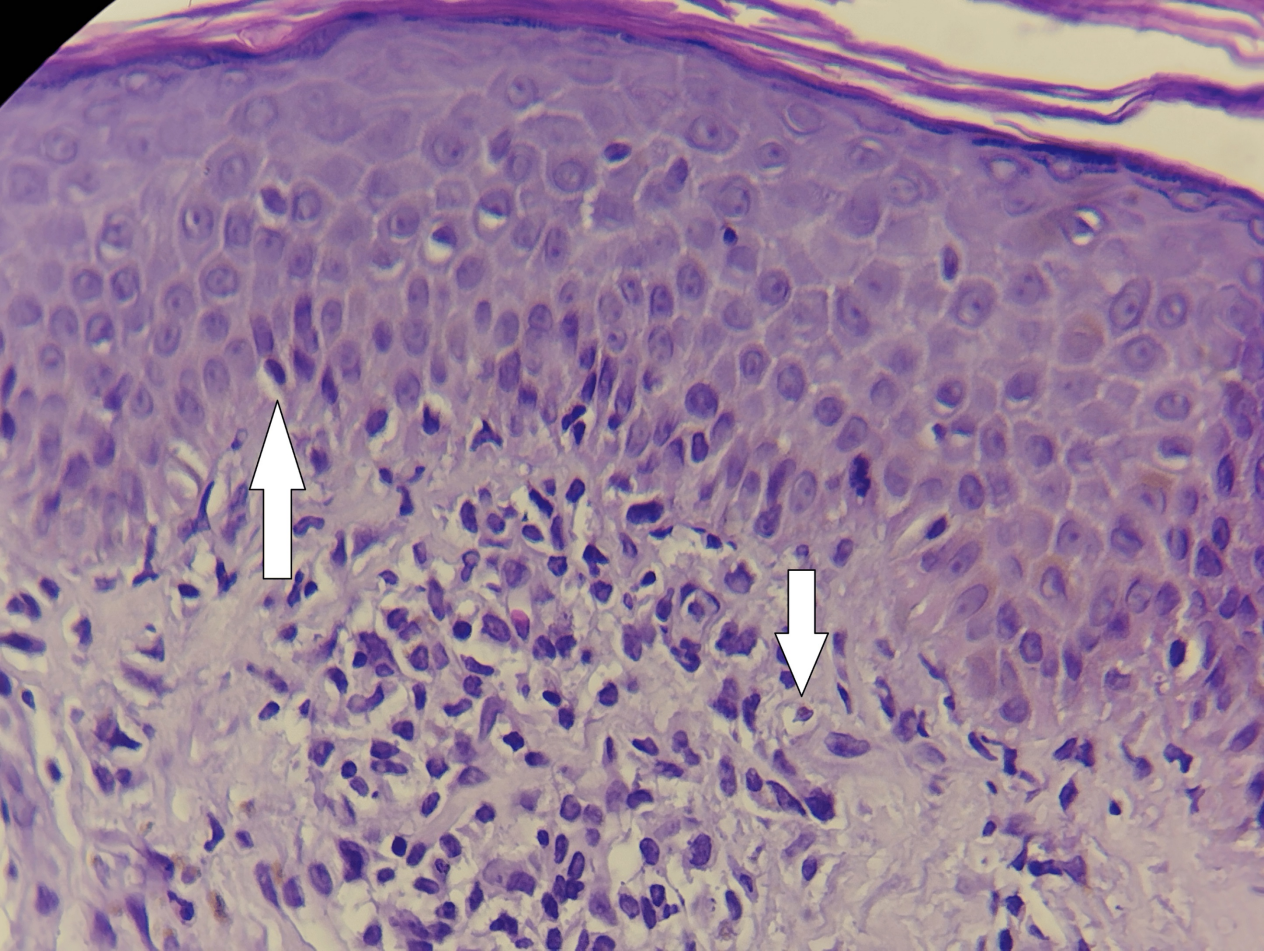
Histopathological findings from a skin biopsy of the right forearm (hematoxylin and eosin stain, 40x). The image shows focal spongiosis in the epidermis and a superficial perivascular inflammatory infiltrate in the dermis, consistent with acute, nonspecific dermatitis. No evidence of malignancy or amyloid deposition was observed.

Vital signs were stable, except for hyperglycemia (glucose 400 mg/dL). Dermatologic examination confirmed diffuse erythroderma, sparing the lower limbs. Laboratory tests showed leukocytosis (12.46 × 10⁹/L) and sustained hyperglycemia, with preserved renal and liver function. A skin biopsy from the right forearm was performed. Histopathological analysis revealed focal spongiosis and features of nonspecific acute inflammation, findings consistent with various etiologies such as eczema, drug-induced dermatitis, or allergic contact dermatitis. Importantly, there was no histological evidence of malignancy or amyloid deposition (Figure 3).

Due to the incomplete drug history and use of unregulated natural products, establishing causality was challenging. The clinical findings did not meet criteria for SJS, TEN, acute generalized exanthematous pustulosis, or drug reaction with eosinophilia and systemic symptoms (DRESS). A final diagnosis of severe drug-induced dermatosis (pharmacodermia) was made.

A comprehensive list of medications was reviewed. The patient had previously received methotrexate, folinic acid, loratadine, and emollients and reported self-medicating with topical products such as Vitacilina® and Barmicil®, along with unspecified herbal remedies. Patch testing was not performed due to the acute presentation and the need for immediate therapeutic intervention. Considering the timing of symptom onset and previous similar episodes, a drug-induced etiology (likely related to the combination of methotrexate and topical agents) was deemed the most probable cause.

Management was conservative, consisting of continued systemic corticosteroids and topical emollients. As the patient remained clinically stable without systemic compromise, immunosuppressants were not necessary. Within seven days, the skin lesions resolved completely without recurrence. Treatment adherence was confirmed, and no corticosteroid-related adverse events were reported.

## Discussion

In older adults, ADRs present considerable diagnostic and therapeutic challenges owing to age-related alterations in pharmacokinetics, polypharmacy, and the presence of multiple comorbidities. Drug-induced dermatoses are common but frequently go undiagnosed in this population, as they often mimic other dermatologic or systemic conditions.

Elderly individuals exhibit decreased renal and hepatic clearance, altered protein binding, and body composition changes that enhance drug accumulation. Polypharmacy further elevates the risk of drug-drug interactions and inappropriate prescriptions. Woo et al. reported that while nonsteroidal anti-inflammatory drugs (NSAIDs) and contrast media are less frequent causative agents in older adults, they are more likely to cause severe ADRs, with odds ratios of 2.16 and 2.09, respectively [5].

Clinical presentations in older adults often differ from those in younger populations. While younger patients tend to develop cutaneous signs, elderly patients may present with visceral symptoms or atypical, muted skin manifestations. Age-related skin changes, including epidermal thinning and diminished immune response, can obscure the identification of dermatologic ADRs.

Wang and Li emphasized that eczema and dermatitis in the elderly may show less pruritus and more xerosis and secondary infections, complicating differential diagnosis [6]. These characteristics underscore the need for comprehensive skin examinations and a high index of suspicion when evaluating new dermatoses in geriatric patients.

In this case, patch testing was not performed due to the acute clinical presentation and the need for prompt systemic therapy. Although the exact etiology could not be confirmed, the combination of methotrexate and topical agents, along with the use of unregulated herbal products, was considered the most likely trigger. The absence of specific information regarding the herbal remedies used limited the ability to apply standard causality assessment tools, such as the Naranjo algorithm or the World Health Organization-Uppsala Monitoring Centre (WHO-UMC) system, further complicating pharmacovigilance efforts.

Our patient’s use of herbal and over-the-counter products complicated causality assessment. The variability and lack of regulation in natural remedies contribute to diagnostic uncertainty, a common challenge in geriatric pharmacovigilance. Herbal products can exert pharmacokinetic and pharmacodynamic interactions, especially in older adults taking multiple medications. These interactions may result in altered drug metabolism via cytochrome P450 modulation or unexpected additive effects, heightening the risk of adverse reactions. Duong et al. highlighted that frailty and comorbidities, such as cardiovascular disease, amplify the severity of ADRs in hospitalized older adults [7].

This case illustrates the importance of obtaining a detailed and inclusive medication history, which must encompass prescription drugs, over-the-counter products, and alternative therapies. Incomplete histories (common in elderly patients) represent a significant barrier to accurate diagnosis and appropriate management. Clinicians must maintain a high level of suspicion when evaluating dermatologic findings in geriatric populations, particularly when unexplained or recurrent.

Prevention and early detection of severe drug reactions in geriatric patients require a multi-pronged approach. Gray et al., in their systematic review and meta-analysis, found that pharmacist-led interventions) such as medication reconciliation, education, and deprescribing (significantly reduced the incidence of ADRs in older adults, particularly when integrated into multidisciplinary care teams [8]. This reinforces the necessity of active surveillance and individualized pharmacotherapy in geriatric care, especially in high-risk patients or those presenting with unexplained dermatologic symptoms.

## Conclusions

This case highlights the diagnostic complexity and clinical relevance of drug-induced dermatoses in older adults. The atypical presentation, compounded by polypharmacy and unregulated natural product use, underscores the need for thorough medication histories and high clinical suspicion. The patient's initial self-medication and the delayed response to outpatient therapy further illustrate how incomplete information can hinder timely diagnosis and management. Conservative management proved effective, emphasizing the importance of individualized, age-appropriate pharmacotherapy. Strengthening pharmacovigilance and promoting multidisciplinary strategies are essential to reduce adverse drug reactions and optimize outcomes in the geriatric population.
